# A Virtual Blind Cane Using a Line Laser-Based Vision System and an Inertial Measurement Unit

**DOI:** 10.3390/s16010095

**Published:** 2016-01-13

**Authors:** Quoc Khanh Dang, Youngjoon Chee, Duy Duong Pham, Young Soo Suh

**Affiliations:** Department of Electrical Engineering, University of Ulsan, Namgu, Ulsan 680-749, Korea; khanhdq8689@gmail.com (Q.K.D.); yjchee@ulsan.ac.kr (Y.C.); duyduongd2@gmail.com (D.D.P.)

**Keywords:** blind, virtual cane, inertial sensor, assisting system, Kalman filter

## Abstract

A virtual blind cane system for indoor application, including a camera, a line laser and an inertial measurement unit (IMU), is proposed in this paper. Working as a blind cane, the proposed system helps a blind person find the type of obstacle and the distance to it. The distance from the user to the obstacle is estimated by extracting the laser coordinate points on the obstacle, as well as tracking the system pointing angle. The paper provides a simple method to classify the obstacle’s type by analyzing the laser intersection histogram. Real experimental results are presented to show the validity and accuracy of the proposed system.

## 1. Introduction

According to an investigation by the World Health Organization, there are 285 million people who are estimated to be visually impaired worldwide: 39 million are blind, and 246 million have low vision as of 2014 [1]. Researchers have been interested in the demand for visually-impaired assistance systems for many years. As a result from Clark-Carter’s research [2], all pedestrians have their preferred walking speed that, for them, is the most physically efficient. Blind pedestrians, when accompanied by a sighted guide, will walk at a speed that is close to that of a sighted pedestrian. However, their walking speed decreases when they walk independently. In other words, the mentality while walking effects their walking performance greatly. With the help of visually-impaired assistance systems, blind people can increase their walking speed and avoid the obstacle in their way. The more information the blind persons have about the environment, the more confidence they have on their walks.

In pedestrian travelling, to explore the environment, ultrasonic sensors [3,4], infrared sensors [5] and cameras [6,7,8] are typically used to help the visually impaired find obstacles within the sensor’s working range. Ultrasonic or infrared sensors can measure the distance from a blind person to an obstacle, but only provide binary information of whether the obstacle exists or not. This limitation makes the system become large if we want to know more detailed information about the obstacle position. For instance, the system in [3] is the combination of five ultrasonic sensors for spectacles and a waist belt, and the system in [5] has three IR sensors attached on a stick and the user’s hat. Most importantly, it is hard to define the obstacle shape with these sensor systems. In another approach, the camera systems [6,7] were successful in finding possible paths for the blind by extracting free spaces from environmental images. However, when an image contains many objects, the extraction may not work accurately. In a similar research, Kim *et al.* [8] use a stereo camera to detect the user’s pointing finger in 3D using color and disparity data, then the object within the estimated pointing trajectory in 3D space is detected, as well as the distance to the object. The drawbacks of this system are its computation cost and limited range of detection. The accuracy depends heavily on the finger gesture process, and the working range is limited in a virtual cylinder around the finger. Another method is using a laser source to investigate the environment, which is the main interest of this paper.

Some related works that use a laser source to scan the environment have been proposed previously [9,10]. Yuan and Manduchi [9] have proposed a system consisting of a laser pointer and a camera, which acts like a virtual white cane, using triangulation, that can measure distances at a rate of 15 measurements per second. The authors used a laser beam and a camera to scan the environment by swinging the system vertically. This could be a simple system with a simple scanning method that analyzes the laser-measured range by the time profile. However, the users need much time to scan the environment. For example, to scan an ascending or descending step, the system needs 2–3 s for a scan, including upward and downward swinging motions. The problem comes from the fact that the laser pointer only provides a single beam. Therefore, to inspect the environment, the user must swing the system vertically, since only a slice of the obstacle is obtained in one swing. Another system that uses a laser source was proposed by Pallejà *et al.* [10]. Light detection and ranging (LiDAR) is used with an accelerometer to investigate the environment in front of the user. The system is installed on the user’s wrist, and the LiDAR points horizontally. The authors considered that the position of the system did not change much while walking. By pointing the system towards the ground, a frontal line created from the intersection of the laser plane and the ground plane is analyzed to estimate the information of the obstacle. The limitation is that only a partial slice of the object is estimated, making it difficult for the user to predict the type of obstacle. Since the system only provides a horizontal laser line with respect to the user, the user has to move forward, so that the laser can scan the whole obstacle. For example, to be informed that there are stairs ahead, the user must walk for at least two seconds, so that the change in the laser distance could be analyzed. This could sometimes be dangerous for the user. Besides, the assumption that the position and the orientation of the system are not changing much while walking could lead to a large error.

To overcome these limitations, we propose an indoor system that includes a laser line, a camera and an inertial measurement unit (IMU). The user scans the environment by swinging a vertical laser line horizontally as the user’s hand movement is estimated using an inertial sensor unit. By scanning, the visually impaired can obtain more information from the environment, which will help them be more confident while walking and increase the chance of avoiding obstacles. Different from [9], our system can provide instant information of the environment instead of swinging a laser beam vertically. Additionally, unlike the system in [10], where the system only provides a slice of the obstacle, our system can provide the information of the obstacle shape while walking.

The main objectives of the system are to classify the obstacles and to estimate the distance from them to the user. The obstacle type is recognized by analyzing the laser projection shape on the obstacle, while the distance to the obstacle is calculated based on the distance of the closest laser point that belongs to the object. From the inertial sensor, the system pointing angle is tracked with respect to the navigation coordinate frame. This estimated pointing angle is combined with the laser stripe on the obstacle’s surface to compute the laser point coordinates in the navigation coordinate frame. If we analyze the histogram of the laser point coordinates by height, the obstacle can be classified into wall, stairs or other obstacles. In addition to this, the obstacle distance is also estimated. To provide this environment information to the visually impaired, simple sound feedback using a single tone that has a volume inversely proportional to the distance to the obstacle can be used. Some voice indications can also be provided to give environmental danger warnings. The advantage of the proposed system is that it provides more information, since a vertical laser line is used to scan the environment horizontally with respect to the user.

This paper is organized into five main parts. Section 2 gives an overview of the proposed system. In Section 3, the obstacle classification and obstacle distance estimation methods are explained in detail. Section 4 describes the experimental procedures and results. Finally, some conclusions are discussed in Section 5.

## 2. System Overview

**Figure 1:**
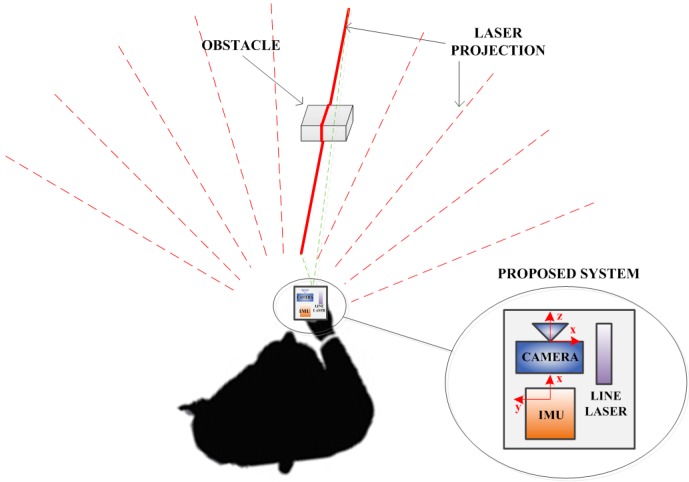
System overview.

The system consists of a camera, a laser line source and an IMU. There are three coordinate systems in the paper that are the camera coordinate frame, the IMU coordinate frame and the temporary world coordinate frame, which will be introduced later. The camera coordinate frame has the origin at the pin hole of the camera; the x and y axes make a plane that is parallel with the camera CCD screen, and the z axis points out and is orthogonal to the CCD screen. The IMU coordinate frame has the xy plane parallel with the sensor’s bottom, and the z axis points up. The IMU is mounted so that its x coordinate axis coincides with the z axis of the camera coordinate frame, as in Figure 1. The laser line generates a laser stripe when intersecting with an obstacle, and this stripe is observed by the camera. Since all devices in the system are fixed, the laser plane equation can be computed and is unique in the camera coordinate frame. Therefore, for each two-dimensional (2D) laser point observed in the camera image frame, its coordinates can be computed in three dimensions with respect to the camera coordinate frame. If we know the position of the system corresponding to the blind person, the three-dimensional (3D) position of the scanned object can be estimated.

The system is held at the blind person’s front and close to the waist (Figure 1). This is a comfortable position, such that the blind person only needs to swing his or her wrist to scan the environment. The laser line is placed so that its projection on the floor is a vertical stripe. When the user swings the system horizontally, the vertical light can scan a large space in front of the user.

The information of the environment is analyzed in each image frame. In each frame, the laser points are extracted based on their intensities, since the laser provides a brighter source than other environmental light sources. A set of laser points is then divided into a basic line and obstacle members. The basic line consists of laser points that lie on the floor, while the obstacle members are the laser point coordinates on the obstacle surface. From this separation, obstacles can be classified into walls, stairs and other obstacles by analyzing their laser shapes. Furthermore, from the laser point coordinates, the distance from the user to the obstacle is also calculated. While the system is swung, its orientation with respect to the navigation coordinate frame can be tracked using a Kalman filter [11]. The estimated orientation is used to approximate the pointing angle of the system in the navigation coordinate frame. It can also be used to detect the case when the user points the system horizontally to turn off the laser (to avoid harmful effects on others’ eyes).

## 3. Virtual Blind Cane Operation

In addition to the camera and IMU coordinate frames, we consider a temporary world coordinate frame whose origin coincides with the IMU coordinate frame origin at the beginning and ending of a swing. The z axis of the temporary world coordinate frame is chosen to be upward, while the x and y axes are arbitrary.

Our goals are to classify the obstacle types and estimate the obstacle distance while swinging the system to scan the environment. To achieve these goals, sensor calibration and initial system height estimation are necessary. While the system is in use, the swing movement is estimated based on the IMU data. This movement is then combined with the laser information computed in the camera coordinate frame to give the 3D coordinates of the laser intersection points with respect to the temporary world coordinate frame. Based on this expression, an obstacle can be recognized by analyzing the laser point histogram, and the distance to the obstacle is computed.

### 3.1. Initial Estimations

Since the system is a combination of multiple sensors, a calibration step to estimate the relative position and orientation of each sensor is required. IMU-camera calibration has been studied many times. In [12], a turntable was used with a robot arm to estimate the relative pose (position and orientation) between the IMU and the camera. Another method used the observed positions and orientation analyzed from the camera while capturing a known checkerboard to compare to the trajectory obtained from the IMU in order to optimize the IMU-camera relationship. This paper used the IMU-camera calibration proposed in [13], since it is easy to use, but still gives an accurate result.

**Figure 2:**
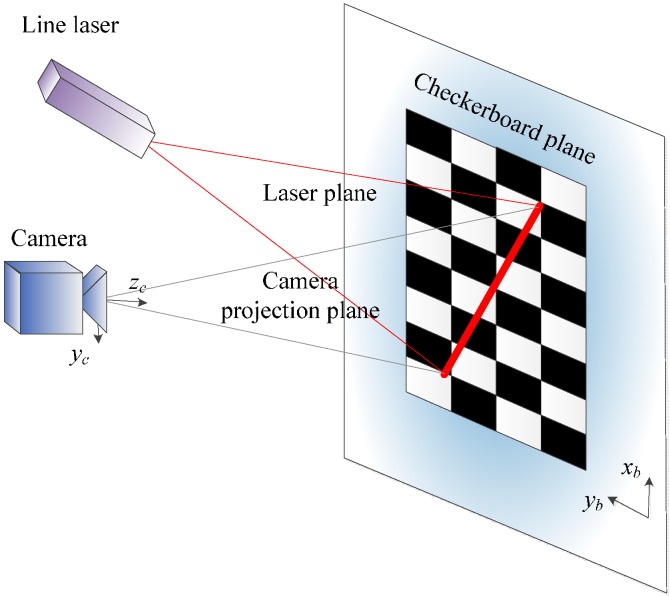
Line-structured light and camera calibration.

The laser plane equation in the camera coordinate frame is obtained in the camera-laser calibration. This calibration consists of capturing a known checkerboard at different positions, as in Figure 2. In each position, two images are taken, where one image contains the laser line and the other does not (the laser is turned off). By subtracting one image from the other, a new image that only contains the laser line is obtained. These laser coordinate points belong to the laser line plane, as well as the planar checkerboard. Since the position and orientation of the checkerboard is known in the camera coordinate frame, the extracted laser point coordinates can be computed with respect to the camera coordinate frame. This method can be found in many research papers about cameras and structured light calibration, such as [14,15].

After the calibration steps, the relationships between the camera, IMU and laser plane have been defined. The camera and line laser now become a unified system, which can be called the line laser-based vision system. This system has a coordinate frame that coincides with the camera coordinate frame. Since all sensors are fixed on a board, their relative positions and orientations are also constant. Therefore, the calibration process only needs to be done once.

The blind person scans the environment by swinging the system horizontally. Based on this fact, it is assumed that the height of the system with respect to the ground does not change much while the system is swinging. This height can be estimated based on the orientation of the IMU and the laser stripe distance.

Before using, to compute the system’s height from the ground, the system is held stably for a few seconds. We note that the height from the ground to the system only needs to be estimated one time before using. In this state, the IMU observes the gravitational force to calculate the rotation matrix from the IMU coordinate frame to the temporary world coordinate frame. Let the temporary world coordinate frame coincide with the navigation coordinate frame; then, we have the rotation matrix $R_{I}^{W}$ from the IMU to the temporary world coordinate frame based on the accelerometer outputs. Let P be the intersection point of the camera coordinate frame z axis and the ground. The distance from the IMU coordinate frame origin to P(${\left[r_{P}\right]}_{W}$) is computed based on the IMU-camera translation $t_{C}^{I}$ and ${\left[r_{P}\right]}_{C}$, where ${\left[r_{P}\right]}_{C}$ is the position of P in the camera coordinate frame. The system height $h_{0}$ is computed as:

(1) $$h_{0}=\left[\begin{array}{ccc} 0 & 0 & 1 \end{array}\right]{\left[r_{P}\right]}_{W}=\left[\begin{array}{ccc} 0 & 0 & 1 \end{array}\right]R_{I}^{W}\left(R_{C}^{I}{\left[r_{P}\right]}_{C}+t_{C}^{I}\right)$$

### 3.2. User’s Swing Analysis

Normally, the walking speed of visually-impaired people is much smaller than normal vision people. The result in [16] showed that the walking speed of a blind person was about 0.4 m/s. Another research work on the association between visual impairment and mobility performance in older adults [17] concluded that the walking speed of visually-impaired people was on average 0.6 m/s. With the help of an aiding system, the visually-impaired people can increase their walking speeds, which range from 0.6 m/s to 0.8 m/s [18].

**Figure 3:**
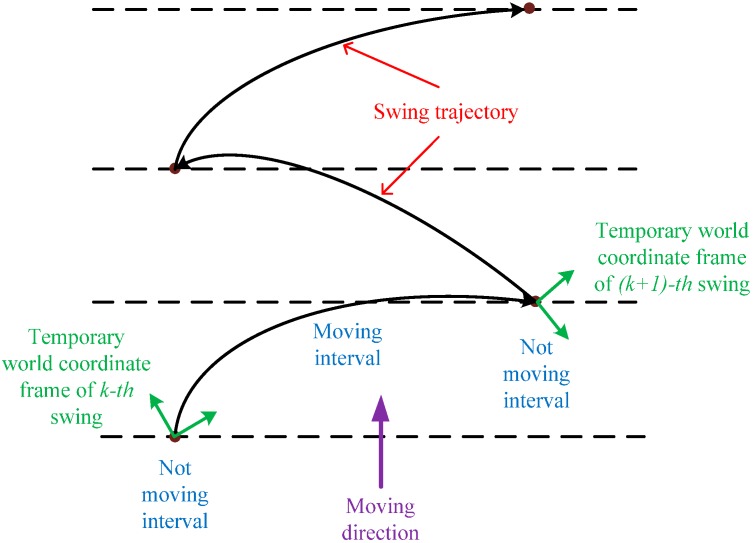
User’s swing motion.

The motion of the blind person’s hand while scanning the environment is described as in Figure 3. This motion consists of a moving interval (swing interval) and two stationary intervals (the beginning and ending of the swing intervals) with respect to the blind person’s body. The motion of the hand is tracked using a Kalman filter with the initial position at the “stationary point” of the k*-th* swing and the stopping point at the (k+1)*-th* swing in the temporary world coordinate frame. The temporary world coordinate frame is reset at each swing (see Figure 3 for details), as well as Kalman filter parameters to avoid accumulated errors. The motion estimation is only considered in each swing separately. In other words, the accumulated error at the k*-th* swing does not affect the (k+1)*-th* swing, since it is reset to zero when the temporary world coordinate frame is reset.

In this system, the IMU has two main roles. Firstly, the stationary interval of the IMU can be detected when gyroscope values for all three coordinates are approximately zero. In this stationary interval, the accelerometer measures the body acceleration and the gravity. Since the movement of the blind person is slow and the acceleration is small during stable walking, it is assumed that the IMU only observes gravity at stationary points. The error given by this assumption was small enough that it does not affect the estimation result much (see [10] for a similar error). Based on this assumption, the orientation of the system to the temporary world coordinate frame at the initial position of each swing can be computed using a triad algorithm [19]. In the second role, the IMU provides the acceleration and angular rate of the system while moving. This information is used in a Kalman filter-based motion tracking algorithm, which uses the system’s initial orientation as the initial value to track the orientation of the system in each swing. Once the orientation of the system is tracked, the pointing angle of the system can also be computed. We note that only the roll and pitch angles are estimated if we observe gravity. However, in this paper, the heading angle is not important since the users intuitively know where their hands are pointing, and only the pitch angle affects the estimation result.

The inertial navigation motion tracking in this paper uses the errors in quaternion, position and velocity as the states of an indirect Kalman filter. The states consist of the errors in $\hat{q}$, $\hat{v}$ and $\hat{r}$ as follows:

(2) $$x\left(t\right)=\left[\begin{array}{c} {\bar{q}}_{e}\\ r_{e}\\ v_{e} \end{array}\right]\in {ℜ}^{9}$$

where “^” denotes estimation and q, v and r stand for the quaternion, velocity and position of the system in the temporary world coordinate frame, respectively.

From the assumption that ${\bar{q}}_{e}$ is small, we obtain the following [20]:

(3) $$\dot{x}\left(t\right)=Ax\left(t\right)+\left[\begin{array}{c} -\frac{1}{2}n_{g}\\ 0\\ -R^{T}\left(\hat{q}\right)n_{a} \end{array}\right]$$

where:

$$A≜\left[\begin{array}{ccc} \left[-y_{g}\times \right] & 0 & 0\\ 0 & 0 & I\\ -2R^{T}\left(\hat{q}\right)\left[y_{a}\times \right] & 0 & 0 \end{array}\right]$$

and $n_{a}\text{ and}\;n_{g}$ are accelerometer and gyroscope noise, respectively. $R\left(\hat{q}\right)$ is the estimated rotation matrix from the IMU coordinate frame to the temporary world coordinate frame, and $y_{g}\;\text{and}\;y_{a}$ are the gyroscope and accelerometer outputs, respectively. Equation (3) is discretized with the sampling period T [11]:

(4) $$x_{k+1}=A_{d,k}x_{k}+w_{k}$$

where $A_{d,k}\approx \exp\left(A\left(kT\right)T\right)$ and $w_{k}$ is a Gaussian white noise with covariance $Q_{d,k}=E\left\{w_{k}w_{k}^{T}\right\}$. Since there is no physical measurement during the motion, the zero velocity during the stationary intervals can be used as a virtual measurement to improve the accuracy of the estimation:

(5) $$z_{zero,k}=H_{zero}x_{k}+n_{v,k}$$

where $z_{zero,k}=\left[0-{\hat{v}}_{k}\right]$ and $H_{zero}=\left[\begin{array}{ccc} 0_{3} & 0_{3} & I_{3} \end{array}\right]$ at the discrete time k with a small Gaussian white noise $n_{v,k}$ with covariance $R_{v,k}=E\left\{n_{v,k}n_{v,k}^{T}\right\}$.

In the case of swinging, it can be assumed that there is no movement at the starting and stopping points of a swing due to the slow and stable walking of the blind person. This assumption can affect the accuracy of estimation. However, the error is still acceptable for detecting the tilt angle of the system and finding separate histogram bins of laser points in the temporary world coordinate frame.

**Figure 4:**
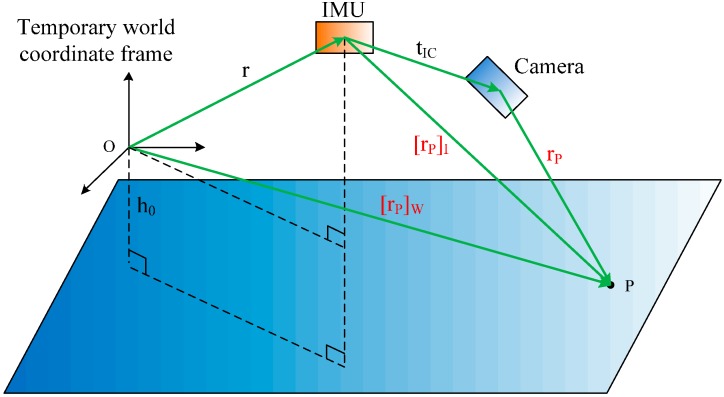
Position updating from laser point coordinates.

We can also utilize the laser information as in Figure 4. The position of a point P in the temporary world coordinate frame in a moving period is:

(6) $${\left[r_{P}\right]}_{W}=C{\left(q\right)}^{T}{\left[r_{P}\right]}_{I}+r$$

where C(q) and r are the rotation from the IMU coordinate frame to the temporary world coordinate frame and the position of the IMU in the temporary world coordinate frame, respectively. Then, ${\left[r_{P}\right]}_{I}$ is the position of point P in the IMU coordinate frame. The height of the IMU is calculated as [21]:

(7) $$\begin{array}{c} h_{0}=\left[\begin{array}{ccc} 0 & 0 & 1 \end{array}\right]{\left[r_{P}\right]}_{W}\\ \;=\left[\begin{array}{ccc} 0 & 0 & 1 \end{array}\right]\left(C{\left(q\right)}^{T}{\left[r_{P}\right]}_{I}+r\right)\;\\ \;=\left[\begin{array}{ccc} 0 & 0 & 1 \end{array}\right]\left(C{\left(\hat{q}\right)}^{T}{\left[r_{P}\right]}_{I}+2C{\left(\hat{q}\right)}^{T}\left[q_{e}\times \right]{\left[r_{P}\right]}_{I}+\hat{r}+r_{e}\right) \end{array}$$

and can be rewritten as:

(8) $$z_{height,k}=H_{height,k}\;x_{k}+n_{z,k}$$

where $z_{height,k}$ is the measurement and $n_{z,k}$ is a measurement noise at the time k:

(9) $$z_{height,k}=h_{0}-\left[\begin{array}{ccc} 0 & 0 & 1 \end{array}\right]\left(\hat{r}+C{\left(\hat{q}\right)}^{T}{\left[r_{P}\right]}_{I}\right)$$

and $H_{height,k}$ is:

(10) $$H_{height,k}=\left[\begin{array}{ccc} 0 & 0 & 1 \end{array}\right]\left[\begin{array}{ccc} -2C{\left(\hat{q}\right)}^{T}\left[{\left[r_{P}\right]}_{I}\times \right] & I_{3} & 0_{3} \end{array}\;\right]$$

### 3.3. Obstacle Classification

**Figure 5:**
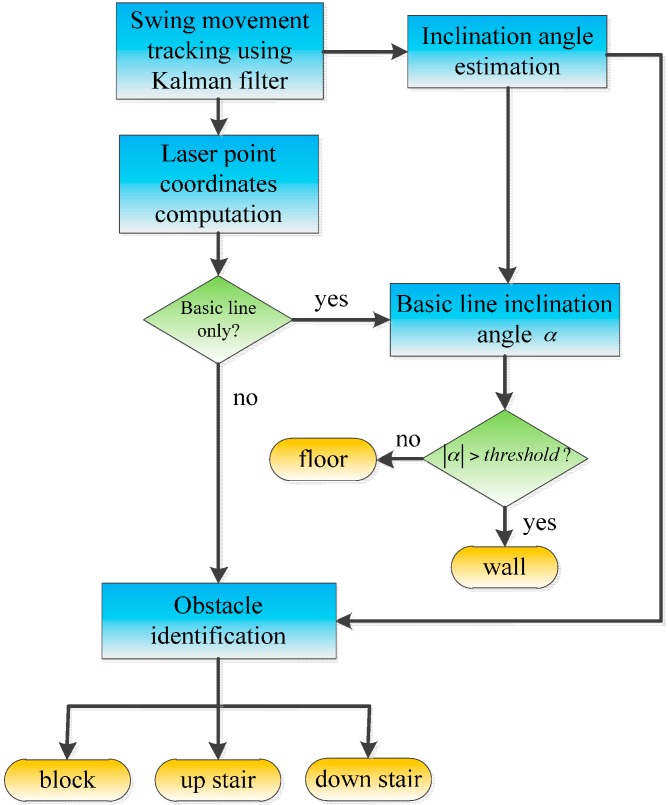
Obstacle classification process.

This part describes the obstacle classification process. It could be assumed that the usual indoor obstacles are walls, stairs and blocks. The obstacle classification process is illustrated in Figure 5. Starting at the “stationary point”, the motion of the hand is estimated using a Kalman filter. The information of interest from the hand motion tracking is the system orientation. From the system orientation, the inclination angle of the hand can be computed. This inclination is used together with the laser information to define an obstacle and is later used in detecting the horizontal pointing pose of the system.

**Figure 6:**
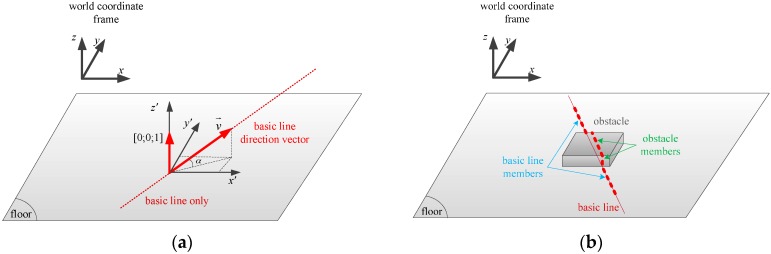
Laser points’ contribution. (**a**) Floor or wall case: only a basic line exists; (**b**) Obstacle case: the laser stripe contains an obstacle and basic line members.

In each camera image, we can separate the laser points into the ground and obstacle members, since the laser point coordinates are known in the camera coordinate frame. By forming a basic line from some of the laser points closest to the user, the laser points can be divided into two cases by comparing their distances to the basic line. The first case is when all of the laser points belong to the basic line (see Figure 6a), while in the second case, the laser points are separated into those belonging to the basic line and those associated with an object (see Figure 6b). When the image only contains the basic line, there is a chance that the entire laser line is on the floor or on a plane, such as a wall or large obstacle. In this case, the inclination angle of the basic line (not in the image, but in the 3D space) is checked to define whether the laser line is on the floor or on a wall. The inclination angle α of the basic line in the temporary world coordinate frame can be calculated from the angle of the basic line direction vector $\vec{v}$ and the z axis unit vector:

(11) $$\alpha =\frac{\pi }{2}-\arccos\left(\left(\vec{v},{\left[\begin{array}{ccc} 0 & 0 & 1 \end{array}\right]}^{T}\right)\right)$$

The basic line direction vector $\vec{v}\;$in the temporary world coordinate frame is the normalization of:

(12) $$v_{W}=C{\left(q\right)}^{\prime }R_{C}^{I}v_{C}$$

where $v_{C}$ is the basic line direction vector in the camera coordinate frame, which is formed by two arbitrary points in the basic line, and C(q) represents a rotation matrix corresponding to q. If the inclination angle α of the basic line is larger than a threshold value, it can be inferred that all of the laser points are on a wall or on a large plane. Otherwise, they lie on the floor. It is easy to define a wall or a floor based on the laser stripe inclination angle since the inclination of a wall is about 90° while a floor’s inclination angle is approximately 0°.

**Figure 7:**
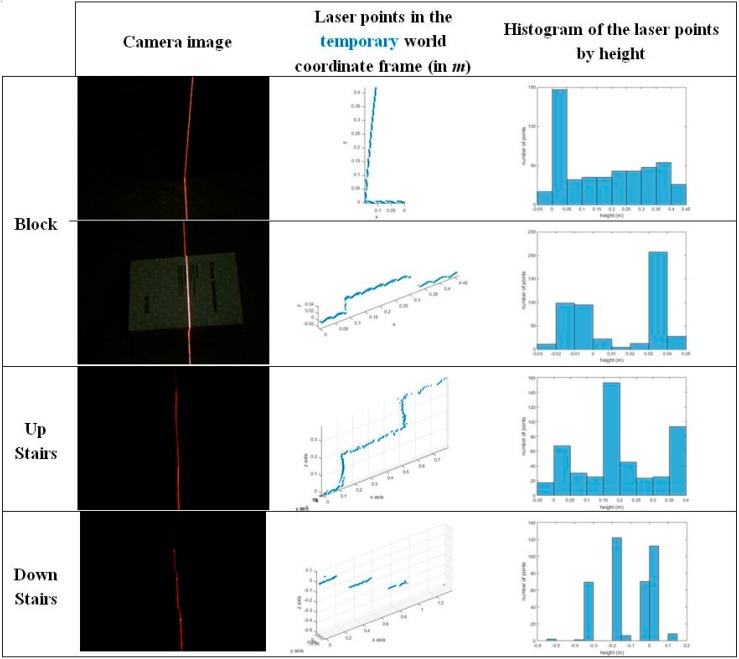
Laser stripe formation on various obstacles.

When some laser points’ distances to the basic line are greater than a threshold, or in other words, the laser points are separated into ground and obstacle members, as in Figure 6b, an obstacle classification process is used to distinguish the obstacles. As shown in Figure 7, different obstacles have different laser intersection shapes. However, they are mainly formed of vertical and horizontal planes, which become laser lines in the camera images. Thus, in the obstacle identification process, we analyze the distribution of the laser points expressed in the temporary world coordinate frame by height to determine an obstacle’s type. Since almost all obstacles in the building, such as blocks and stairs, are formed by vertical and horizontal surfaces, different obstacles provide different histogram forms, as in Figure 7.

The positions of the laser points can be obtained in the IMU coordinate frame, since the relative pose (position and orientation) between the IMU and the camera is known. Once the pose of the IMU has been tracked using an inertial navigation algorithm, the laser point coordinates can be expressed in the temporary world coordinate frame. In Figure 7, to make the histogram analysis by height have an easier view, all of the laser point coordinates are shifted so that the first point of the basic line is at the origin of the temporary world coordinate frame (see the “laser points in the temporary world coordinate frame” column). In the obstacle identification process, the algorithm finds separate histogram bins whose values are greater than a threshold. In other words, the algorithm divides the laser points into groups based on theirs height values. We assume that if a bin’s value (number of points) is greater than a threshold, it represents a horizontal plane that forms the obstacle. After finding histogram bins, two bins are considered to be one bin if they stand next to each other. For example, in Figure 7, in the “down stairs” case, if a threshold of 60 points is set, the histogram bins of laser points that are greater than the threshold are Bins 3, 4, 6 and 7. Since Bin 6 stands next to Bin 7 in the height axis, they are considered to be a single bin; while Bins 3 and 4 are split to be two separate bins. Therefore, the total number of separated bins that can be found in the laser histogram is three. This means that the obstacle is formed by three horizontal planes. We can conclude that it is stairs.

Generally, a block is detected with one or two separate bins, as shown in Figure 7, while stairs normally have three separate bins. In some cases, the histogram bin number for stairs can be two. In these cases, the height of the last laser point with respect to the blind person is compared to the height values of two bins to determine whether the scanned object is stairs or a block. Up or down stairs have a final laser point height much larger or smaller, respectively, than the bin’s height.

### 3.4. Distance to the Obstacle

**Figure 8:**
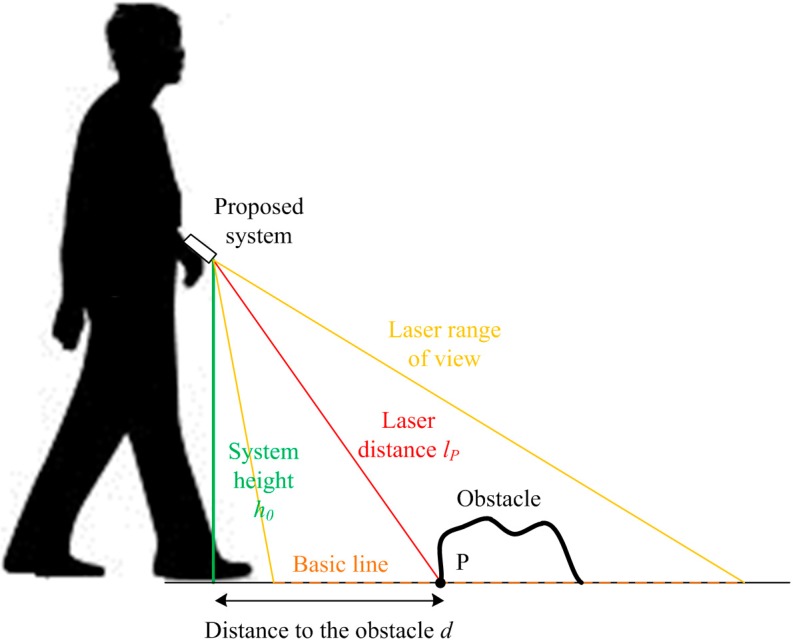
Distance estimation for the obstacle.

In addition to the obstacle identification, the other important information from the environment that needs to be determined is the distance from the obstacle to the blind person. The distance d from the system to the obstacle is computed based on the system height $h_{0}$ and the laser distance $l_{P}$, as depicted in Figure 8. In this system, we chose 40 mm as the threshold for determining whether a laser point belongs to the basic line or not. Assuming that the point P in Figure 8 represents the first point of the obstacle from the view of the blind person, we can determine P as the closest laser point to the blind person that does not lie on the basic line. Since the laser distance $l_{P}$ can be derived from the coordinates of P in the IMU coordinate frame, the distance d can be calculated as:

(13) $$d=\sqrt{l_{P}^{2}-h_{0}^{2}}$$

## 4. Experiments and Results

**Figure 9:**
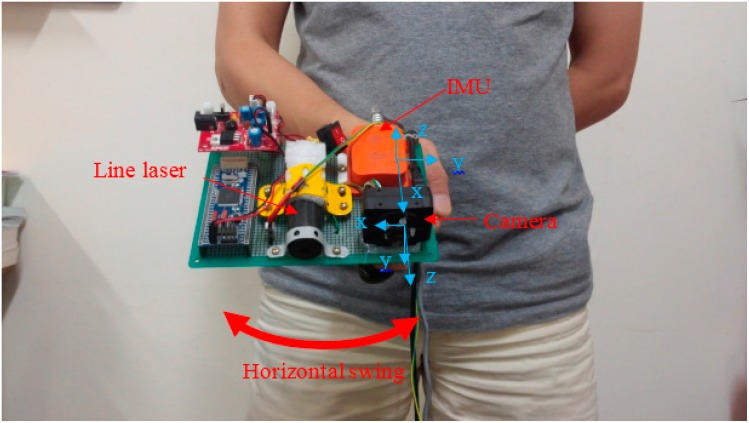
Experimental system.

The experimental setup is shown in Figure 9. The system consists of a Firefly camera, an Xsens IMU and a laser line projector. The camera and the IMU are synchronized using a microprocessor. For processing, a 2.09-kg HP ProBook 6460 b laptop with a configuration of Core i5 2.5 GHz CPU, 4 GB RAM, was used with this experimental system. The experimental system weight is about 250 g. In the experiments, the laptop was carried by a volunteer, while the proposed system was held by hand in front of the volunteer and close to his waist.

### 4.1. System Height Estimation Experiment

Since the system height is used to compute the distance from the user to the obstacle, it is necessary to estimate the system height. To verify the accuracy of the system height estimation with respect to the ground, the system was pointed toward a flat floor at different heights and orientations in each recording process. The position of the system was first calculated based on the method proposed in Section 3.1 and was then compared to the ground truth value tracked by the OptiTrack system [22]. The experiment results verifying the IMU’s height estimation are given in Table 1.

During operation, the system height can be changed a certain amount. However, this variation affects the distance estimation only moderately. For example, if the system is held close to the user’s belt, the system height could be around 1 m. With a normal pointing angle of 45° with respect to the horizontal plane, the laser distance could be around 1.4 m. If the system height is changed by 10 cm, then the distance error from the user to the obstacle is smaller than 10 cm. This is an acceptable error compared to the walking distance.

**Table 1:** Height estimation by the IMU.

Number of Experiment	Ground Truth Height (cm)	Estimated Height (cm)	Error (cm)
**1**	66.50	66.37	0.13
**2**	71.00	71.40	0.40
**3**	75.00	75.00	0.00
**4**	80.00	80.43	0.43
**5**	82.00	81.99	0.01
**6**	85.50	85.96	0.46
**7**	90.00	89.94	0.06
**8**	92.00	92.16	0.16
**9**	95.00	94.41	0.59
**10**	100.00	99.51	0.49
**Average Error (cm)**			0.273
**Standard Deviation (cm)**			0.193

### 4.2. Obstacle Classification Experiment

**Figure 10:**
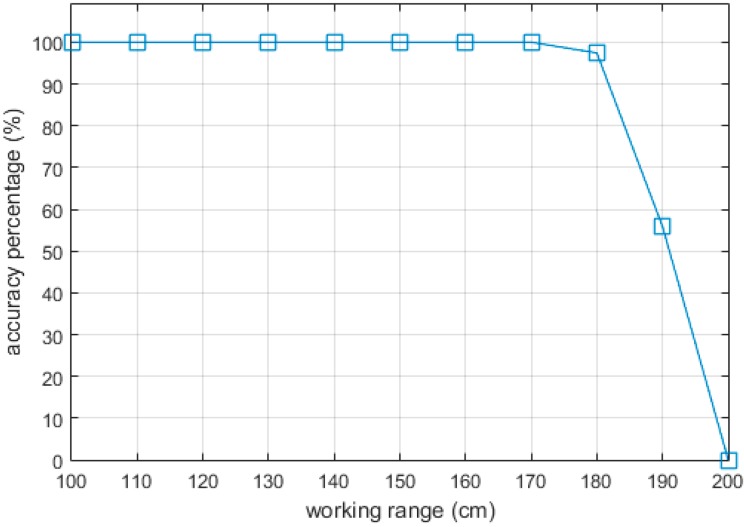
Working range accuracy.

In this experiment, the proposed algorithm was tested in offices and corridors with typical obstacles, such as chairs, tables, walls and stairs. With the current configuration, the effective working range of the system was about 1.8 m from the user, because the laser point extraction was affected by the environmental illumination. We conducted an experiment to find the effective working range of the system. The distance from the object to the system was changed after the object classification process was completed. The results are given in Figure 10, which shows the relationship between the distance from the system to the object and the successful classification percentage.

The working range is also affected by the baseline between the camera and the line laser due to the error given by a small parallax. To verify the accuracy of the object classification of the system using the current configuration, an experiment was carried out with three boxes with heights of 4, 8 and 13.5 cm, respectively. Boxes were captured by the system at different distances ranging from 1 m to 1.9 m. The laser stripes on box surfaces were analyzed to estimate the box’s heights, which, in other words, represents the change in laser point coordinates. The accuracy of the system (in cm) is shown in Table 2.

**Table 2:** A result of estimating different obstacle heights (in cm) at different distances (in m).

		Distance										Mean	Error
		1 m	1.1 m	1.2 m	1.3 m	1.4 m	1.5 m	1.6 m	1.7 m	1.8 m	1.9 m		
**Object (cm)**	4	4.76	4.81	4.04	4.13	4.69	5.03	4.78	4.59	5.01	4.50	4.63	0.63
	8	8.70	8.14	8.04	7.60	7.69	8.06	9.53	7.86	9.57	9.18	8.44	0.44
	13.5	12.70	11.77	11.07	12.32	11.35	13.35	14.03	13.00	14.34	13.44	12.74	0.76

The result indicates that with the proposed configuration, the system can recognize obstacles with the current camera-laser baseline. Increasing the baseline of the camera and the laser will provide a better object estimation. However, the limitation is that the size of the system will be increased. As a handheld device, we chose this baseline to balance the object estimation error and the size of the system.

A volunteer was asked to scan the environment while walking. The experiment was repeated five times for each environment. The sampling rate of the camera is 20 Hz. We note that all experiments were done within the effective working range of the system. The laser shapes on the obstacles were analyzed, and the classification results are given in Table 3, in which the accuracies are expressed as percentages. Table 3 shows that the system can accurately recognize walls and floors. Other obstacles, such as stairs and a block, could also be detected with a high accuracy of up to 96%.

Since the wall and floor only contained the basic line, they were the easiest to recognize. The obstacles used in the room experiment were blocks, which were formed by one vertical and two horizontal planes. Therefore, sometimes, they were recognized as stairs. For the stairs case, when the laser stripe was not fully captured, the laser point histogram only contained two separate bins, causing the stairs to be classified as a block.

**Table 3:** Obstacle classification accuracy (%) within the effective working range (1.8 m).

		Real Features				
		Wall	Floor	Up Stairs	Down Stairs	Block
**Number of Samples (Images)**		1315	753	846	1101	785
**Detected as (%)**	**Wall**	100	0	0	0	0
	**Floor**	0	100	0	0	0
	**Up Stairs**	0	0	96.27	0	3.85
	**Down Stairs**	0	0	0	96.11	0
	**Block**	0	0	3.73	3.89	96.15

### 4.3. Distance to Obstacle Estimation

The distance to the obstacle estimation experiment was set up with a block lying on a flat floor. Before this experiment, the system height was estimated. Then, the user swung the system to scan the environment while standing in certain marked positions. The position of the block was marked on the floor, and the distance from the user to the block was measured directly. These ground truth distances were used in comparison to the estimated distances calculated based on Section 3.4. Table 4 summarizes the ground truth and estimated distances.

The maximum error was 11.15 cm, while the minimum error was 4.08 cm. These errors are acceptable compared to the distance of a normal stride. The mean error of 6.17 cm shows that the system is feasible for real life application.

**Table 4:** Estimation of distance to the obstacle.

Number of Experiment	Ground Truth Distance (cm)	Estimated Distance (cm)	Error (cm)
**1**	60	55.92	4.08
**2**	75	70.15	4.85
**3**	90	84.38	5.62
**4**	105	101.02	3.98
**5**	120	126.95	6.95
**6**	135	139.50	4.5
**7**	150	141.97	8.03
**8**	165	171.33	6.33
**9**	180	191.15	11.12
**Average error (cm)**			6.17
**Standard deviation (cm)**			2.18

### 4.4. Dynamic Experiment with the OptiTrack Camera System

In this experiment, the volunteer scanned the environment with the system while the system position was observed by the OptiTrack cameras. A box was placed in a known location with respect to the OptiTrack camera system’s coordinate frame. Since the purposes of the system are to recognize the obstacle’s type and to estimate the distance from the user to it, an OptiTrack marker was attached to the IMU to record the system position. Once the marker’s position was known, the distance from the marker (also the system) to the box was computed. This ground truth value was compared to the distance that was estimated by the system based on Section 3.4.

**Figure 11:**
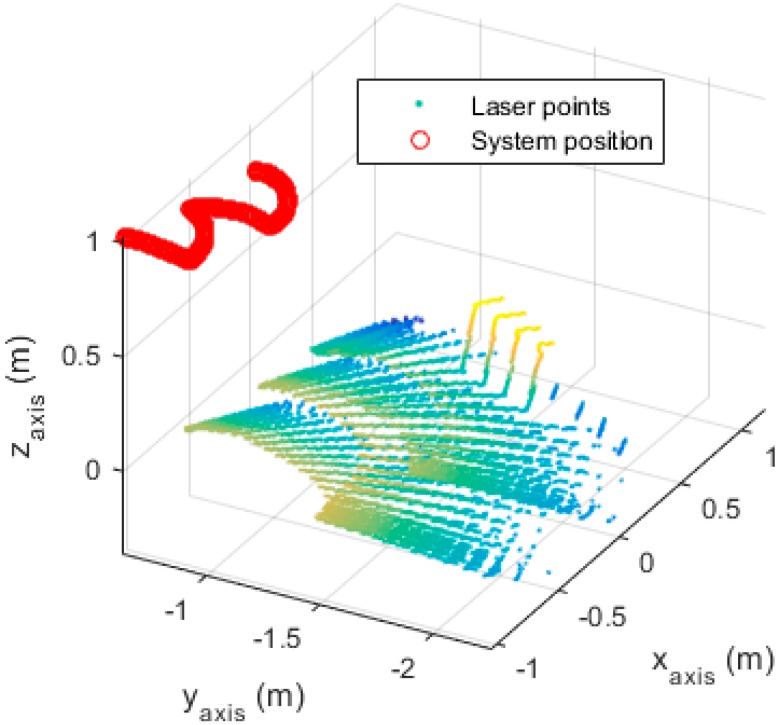
Results of the dynamic experiment.

The overview of the experimental results is given in Figure 11, which shows the positions of the system and the corresponding laser stripes. The system scanned the floor until it met a box. There were 136 images captured, where eight images contained the box, and the rest (129 images) were floor images. The system successfully recognized the box in seven images (87.5%) and the floor in 129 images (100%). One image was incorrectly classified as a down stair case.

The distance from the obstacle to the system was also computed, based on the images that contained the box. Compared to the real distances, which were obtained from the marker’s position relative to the box, the estimated distances had an average error of 11.58 cm; the best estimation was 2.79 cm, and the worst estimation was 22.36 cm.

Besides, the assumption that $h_{0}$ is not changing much while swinging is also shown in Figure 11. As can be seen from the figure, the system height, which is observed by the OptiTrack system, does not change much. The height at the starting point is 1.012 m, while the height at the ending point is 1.018 m. The minimum height value is 0.9837 m, and the maximum height value is 1.028 m.

## 5. Discussion

This paper proposes a blind navigation assistance system consisting of an IMU, a laser line and a camera. The objectives of the system are to recognize an obstacle and to find the distance from the user to the obstacle. The obstacle’s type is classified by investigating the laser point distribution by height, while the distance to the obstacle is computed by combining the swing movement analysis with the 3D laser point coordinates. Some experiments have been done to verify the accuracy, as well as the feasibility of the system. The results showed that the obstacles can be classified accurately, and the distance to the obstacle has a small error. When the system is applied in a real environment, the estimated distance error is acceptable compared to a normal walking step. The advantages of the system are its simplicity, low cost and accuracy. It can be easily implemented by attaching a line laser to a smartphone or other PDA devices, which already have a built-in IMU and a camera.

However, the limitation of the system is that it is easily affected by strong illumination. Therefore, the system working range is limited, as shown in Figure 10. This led to some large errors in estimating the distances to the obstacles in the dynamic experiment, because some laser points are out of effective working range while the system is swinging. This limitation can be reduced by applying some laser filters or using a stronger laser line.

Another limitation of the system is that the laser light is harmful for the eyes. To avoid this danger, the system gives some indications when the user points the system horizontally. To do this, the system pointing angle is tracked by a Kalman filter, as mentioned in Section 3.2. Although there is an error in the system pointing angle estimation due to the swing of the hand and the movement of the body, the horizontal and ground pointing angles are much different (about 90° for horizontal pointing and about 40° for ground pointing). Therefore, the estimated system pointing angle is used to identify the horizontal pointing case.

At the starting point of a swing, the system pointing angle ρ of the system is calculated based on Equation (55) in [23]:

(14) $$\rho =\frac{\pi }{2}-arccos\frac{a_{z}}{\sqrt{a_{x}^{2}+a_{y}^{2}+a_{z}^{2}}}\;$$

where $a={\left[\begin{array}{ccc} a_{x} & a_{y} & a_{z} \end{array}\right]}^{T}$ is the accelerometer output at that moment. The system pointing angle ρ is then tracked in each swing movement to give the system pointing angle at every sampling point in the swing.

**Figure 12:**
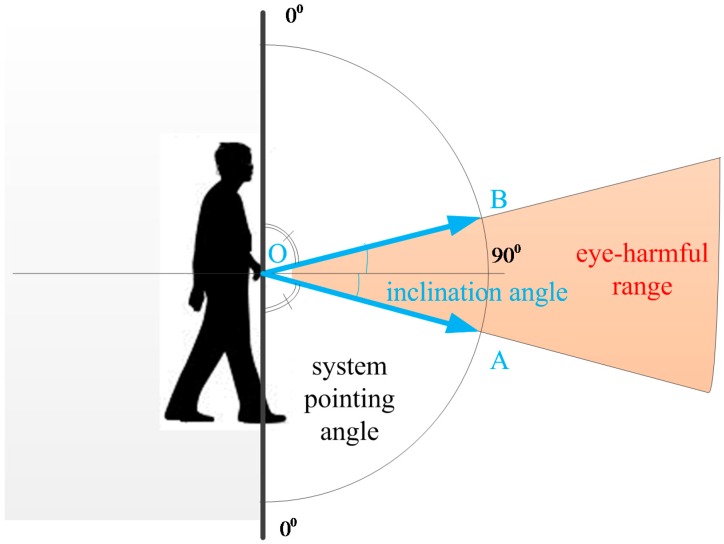
System pointing angle.

Since the z axis of the IMU coordinate frame is pointing upward and its xy plane is parallel to the horizontal plane, the value of the inclination angle, which is computed based on Equation (55) in [23], varies from 90° (when the system is pointed orthogonally to the ground or upward) to 0° (when the system is pointed horizontally). Therefore, we used Equation (14) to express the system pointing angle by subtracting the inclination angle from π2 to have the pointing angle ranging from 0° to 90°. A demonstration is given in Figure 12 below (note that cos(α)=cos(−α); thus, inclination angles in the case of the OA and OB directions have the same value).

**Figure 13:**
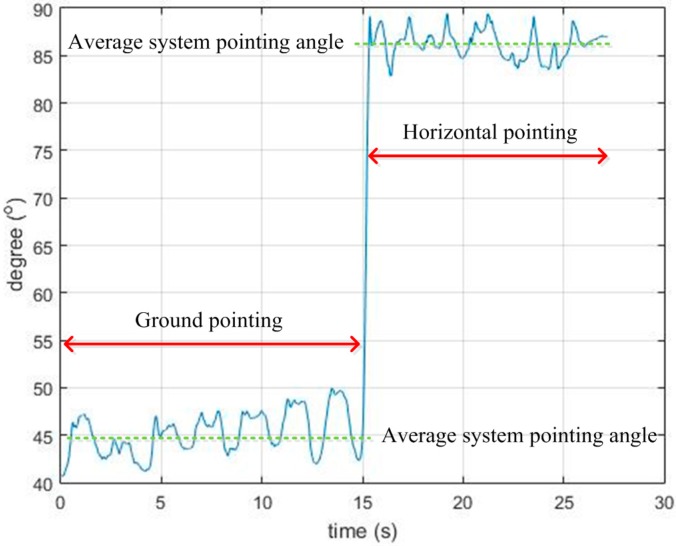
System pointing angle estimation example.

As an example, in Figure 13, the user was walking while scanning the ground from zero to 15 s before the system swung horizontally from 15 to 25 s. As shown in Figure 13, the tracked system pointing angle of the ground scanning period was about 45°, while in the second period, the horizontal swing angle was about 87°. With a large change, the difference between the ground and horizontal swinging can be easily recognized. The system turns off the laser when it detects horizontal pointing.

**Figure 14:**
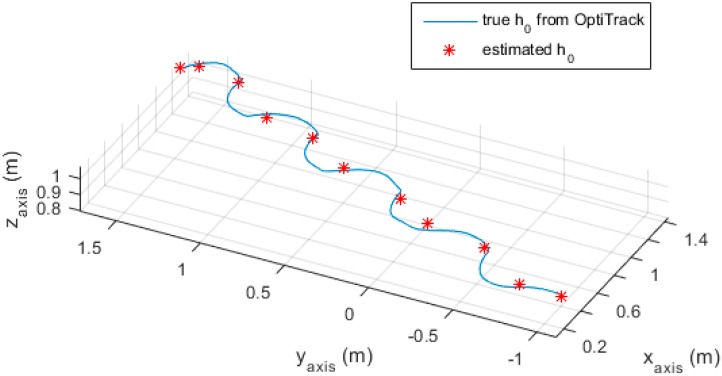
Estimated and true $h_{0}$.

The last thing to discuss is the system height. There is an assumption that the system height does not change much while walking ,since the system is held in front of the users, close to their waist, which is the most comfortable place for long holding and similar to the usual blind cane position. An experiment was done within the range of the OptiTrack system view, where a volunteer used the proposed system while a marker was attached on the system to observe the system height $h_{0}$. The $h_{0}$ at each stationary point in this experiment can be calculated based on the inclination angle of the system and the laser distance. The result is given in Figure 14.

As can be seen from Figure 14, the estimated $h_{0}$ is close to the true value obtained from the OptiTrack camera system. The error between estimated $h_{0}$ and the true value is given in Table 5 below.

**Table 5:** Estimated and true $h_{0}$ at stationary points.

Stationary Point Order	1	2	3	4	5	6	7	8	9	10	11
**True** $h_{0}$ **(m)**	1.0009	1.0178	1.0267	1.0356	1.0196	1.0350	1.0399	1.0442	1.0439	1.0395	1.0362
**Estimated** $h_{0}$ **(m)**	1.0104	1.0395	1.0791	1.0795	0.9772	1.0553	1.0499	1.0167	1.0435	1.0277	1.0377
**Absolute Error (m)**	0.0095	0.0217	0.0524	0.0439	0.0424	0.0203	0.0100	0.0275	0.0004	0.0118	0.0015
**Absolute Mean Error (m)**	0.0219										

The maximum absolute error was about 5 cm, while the average absolute error was 2.2 cm. This result implied that the system height does not change much during walking.

Since the system is used by blind people, sound feedback can be selected to give the indications to the users. Sound feedback has been studied for many years. A well-known sound feedback system is the image to sound technique, which was proposed by Meijer in 1992 [24]. In this method, an image is scanned by the pixel column. In each column, one pixel row position corresponds to a frequency, while the sound amplitude is proportional to the pixel color. This representation provides a sound combined from many frequencies and their own amplitudes. The audience can visualize an image by listening to the column sound, which moves from the left to the right of the image. However, to use this system, the user needs to be trained, and the continuous sounds may disturb the user.

In this system, a simple feedback method can be used. The feedback includes sound feedback and voice description. The sound feedback presented here is a single tone sound with a magnitude proportional to the distance from the blind person to the object, while the voice description gives an indication for some special cases, such as “up stairs two meters ahead”, “horizontal pointing”, *etc.* A higher tone indicates that the user is closer to the obstacle. To avoid sound annoyance, the sound only exists when the system detects certain obstacles.
